# Radiomics feature as a preoperative predictive of lymphovascular invasion in early-stage endometrial cancer: A multicenter study

**DOI:** 10.3389/fonc.2022.966529

**Published:** 2022-08-18

**Authors:** Xue-Fei Liu, Bi-Cong Yan, Ying Li, Feng-Hua Ma, Jin-Wei Qiang

**Affiliations:** ^1^ Department of Radiology, Jinshan Hospital, Fudan University, Shanghai, China; ^2^ Department of Diagnostic and Interventional Radiology, Shanghai Jiao Tong University Affiliated Sixth People’s Hospital, Shanghai, China; ^3^ Departments of Radiology, Obstetrics & Gynecology Hospital, Fudan University, Shanghai, China

**Keywords:** endometrial cancer, lymphovascular space invasion (LVSI), magnetic resonance imaging, radiomics, nomogram

## Abstract

**Background:**

The presence of lymphovascular space invasion (LVSI) has been demonstrated to be significantly associated with poor outcome in endometrial cancer (EC). No effective clinical tools could be used for the prediction of LVSI preoperatively in early-stage EC. A radiomics nomogram based on MRI was established to predict LVSI in patients with early-stage EC.

**Methods:**

This retrospective study included 339 consecutive patients with early-stage EC with or without LVSI from five centers. According to the ratio of 2:1, 226 and 113 patients were randomly assigned to a training group and a test group, respectively. Radiomics features were extracted from T1-weighted imaging (T1WI), T2-weighted imaging (T2WI), contrast-enhanced (CE), diffusion-weighted imaging (DWI), and apparent diffusion coefficient (ADC) maps. The radiomics signatures were constructed by using the Least Absolute Shrinkage and Selection Operator (LASSO) algorithm in the training group. The radiomics nomogram was developed using multivariable logistic regression analysis by incorporating radiomics signatures and clinical risk factors. The sensitivity, specificity, and AUC of the radiomics signatures, clinical risk factors, and radiomics nomogram were also calculated.

**Results:**

The individualized prediction nomogram was constructed by incorporating the radiomics signatures with the clinical risk factors (age and cancer antigen 125). The radiomics nomogram exhibited a good performance in discriminating between negative and positive LVSI patients with AUC of 0.89 (95% CI: 0.83–0.95) in the training group and of 0.85 (95% CI: 0.75–0.94) in the test group. The decision curve analysis indicated that clinicians could be benefit from the using of radiomics nomogram to predict the presence of LVSI preoperatively.

**Conclusion:**

The radiomics nomogram could individually predict LVSI in early-stage EC patients. The nomogram could be conveniently used to facilitate the treatment decision for clinicians.

## Introduction

Endometrial cancer (EC) is the most common gynecologic malignancy in developed countries (1). Depth of myometrial invasion, tumor grade, and histologic subtype are the known factors to affect EC prognosis. Recent studies showed that the presence of lymphovascular invasion (LVSI) was an independent risk of prognostic factors of EC (2). LVSI is strongly associated with high risk of recurrence and poor survival rate in early-stage EC (3). A previous study showed that LVSI was an independent risk factor for developing pelvic lymph node metastasis in early-stage EC patients (4). Thus, it is important to determine the presence of LVSI preoperatively for the decision-making whether to perform lymphadenectomy (5). Furthermore, adjuvant treatment is recommended in early-stage EC patients with LVSI (high intermediate risk) (6, 7).

The presence of LVSI is based on the histological diagnosis, which is considered as the tumor cells deposit in the vascular and lymphatic channels of the uterine specimen (3). However, the diagnosis of LVSI could only be referred by the hysterectomy specimen (8). LVSI status is rarely assessable using biopsy specimens. Moreover, Kumar et al. suggested that intraoperative frozen section (IFS) analysis had poor sensitivity for the determination of LVSI, compared to the final hysterectomy specimen (9).

Magnetic resonance imaging (MRI) is a non-invasive technique with high resolution for soft tissue. Functional MRI was previously used to predict LVSI in EC patients. However, limited information could be provided to assess the presence of LVSI preoperatively (10). Ueno et al. showed a moderate diagnosis performance in preoperatively predicting LVSI based on 2D tumor texture features (11). However, 3D analyses were more representative for the tumor heterogeneity, which might yield better prognostic information (12).

Radiomics, which converted MR images to mineable data in a high-throughput process, may offer abundant information of EC (13). Radiomics has been proposed as a tool for the accurate diagnosis, preoperative risk stratification, or assessment of treatment response in several cancer types (14). Radiomics nomograms integrating radiomics and clinical information were reported able to predict LVSI in breast cancer (15).

We assumed that radiomics could be used to predict the presence of LVSI preoperatively. Thus, in this study, we investigated whether radiomics nomograms integrating radiomics and clinical risk factors could predict the presence of LVSI preoperatively to evaluate the aggressiveness in early-stage EC.

## Materials and methods

### Patients

**Figure 1:**
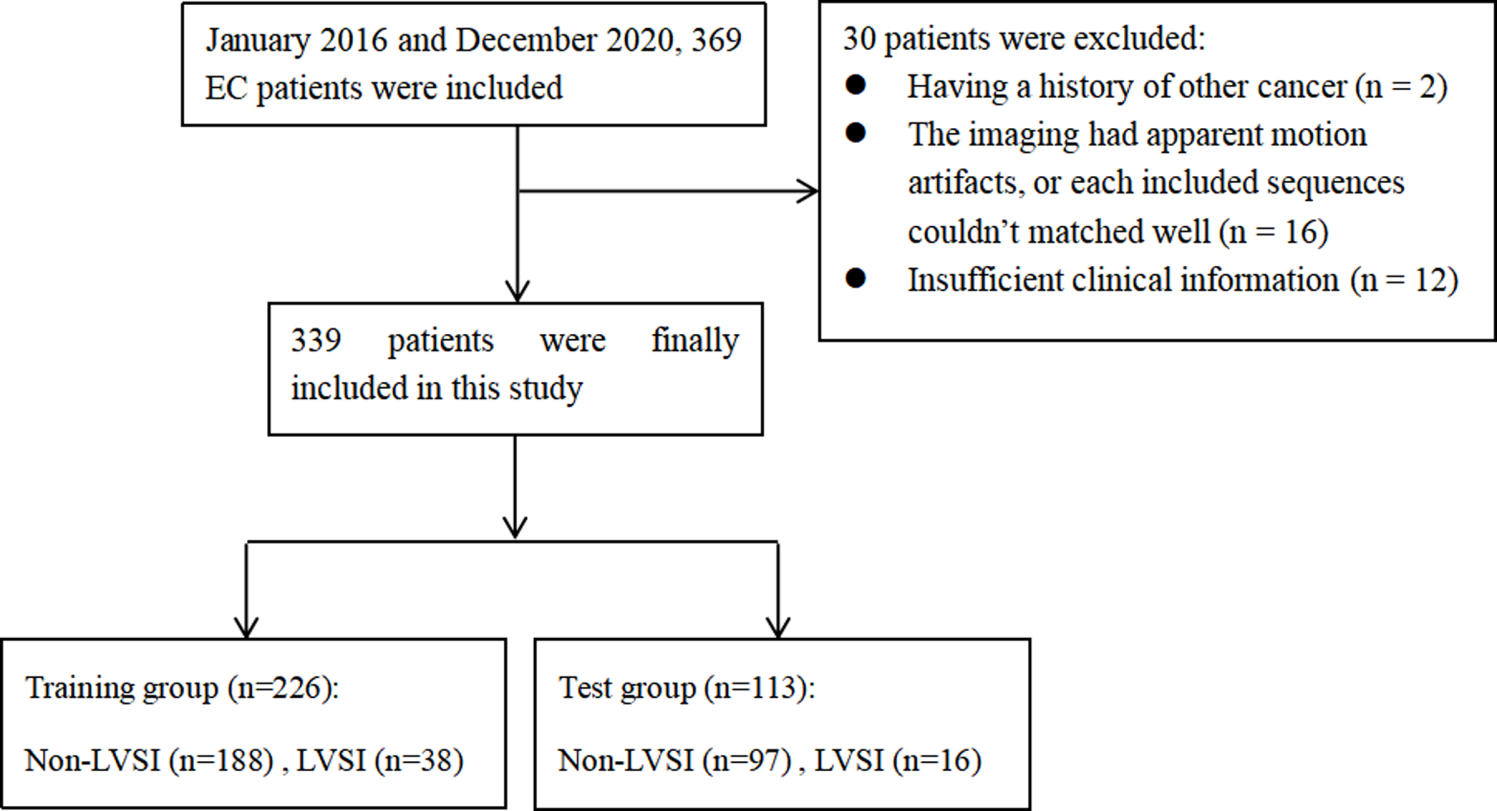
The work flow of this study.

This retrospective study obtained the approval of the local institutional review board (No. 2020-10), and the requirement for informed consent was waived. The multicenter study was performed jointly by five centers: centers A, B, C, D, and E. Three hundred sixty-nine patients from the five centers were collected consecutively during January 2016 to December 2020, and all the patients had MR imaging examination before the treatment for EC. The included criteria were as follows: 1) histological diagnosed stage I EC; 2) basic MRI sequences including T1-weighted imaging (T1WI), T2-weighted imaging (T2WI) with fat saturation, diffusion-weighted imaging (DWI), apparent diffusion coefficient (ADC), and contrast-enhanced (CE) T1WI sequences; 3) the interval time between MRI examination and surgical less than 30 days. The exclude criteria were as follows: 1) patients with a history of other cancer (n = 2); 2) the imaging had apparent motion artifacts, or the included sequences could not match well (n = 16); 3) insufficient clinical information (n = 12). The patients from all the five centers were randomly divided into a training group (n = 226) and a test group (n = 113) in a ratio of 2:1. Clinical parameters, including age, tumor size, and cancer antigen 125 (CA125), were obtained through the review of clinical data. The workflow is shown in **Figure 1**.

### MRI performance

MRI was performed by using 3.0/1.5-T scanners with a phased-array abdominal coil. The patients lay in a supine position and breathed freely during the acquisition. The following sequences were obtained: T1WI, T2WI, DWI, and CE-T1WI after intravenous administration of gadopentetate dimeglumine at a dose of 0.2 mmol/kg of body weight in a rate of 2 to 3 ml/s. The scanning details are shown in **Supplementary Table 1**.

### Image segmentation

**Figure 2:**
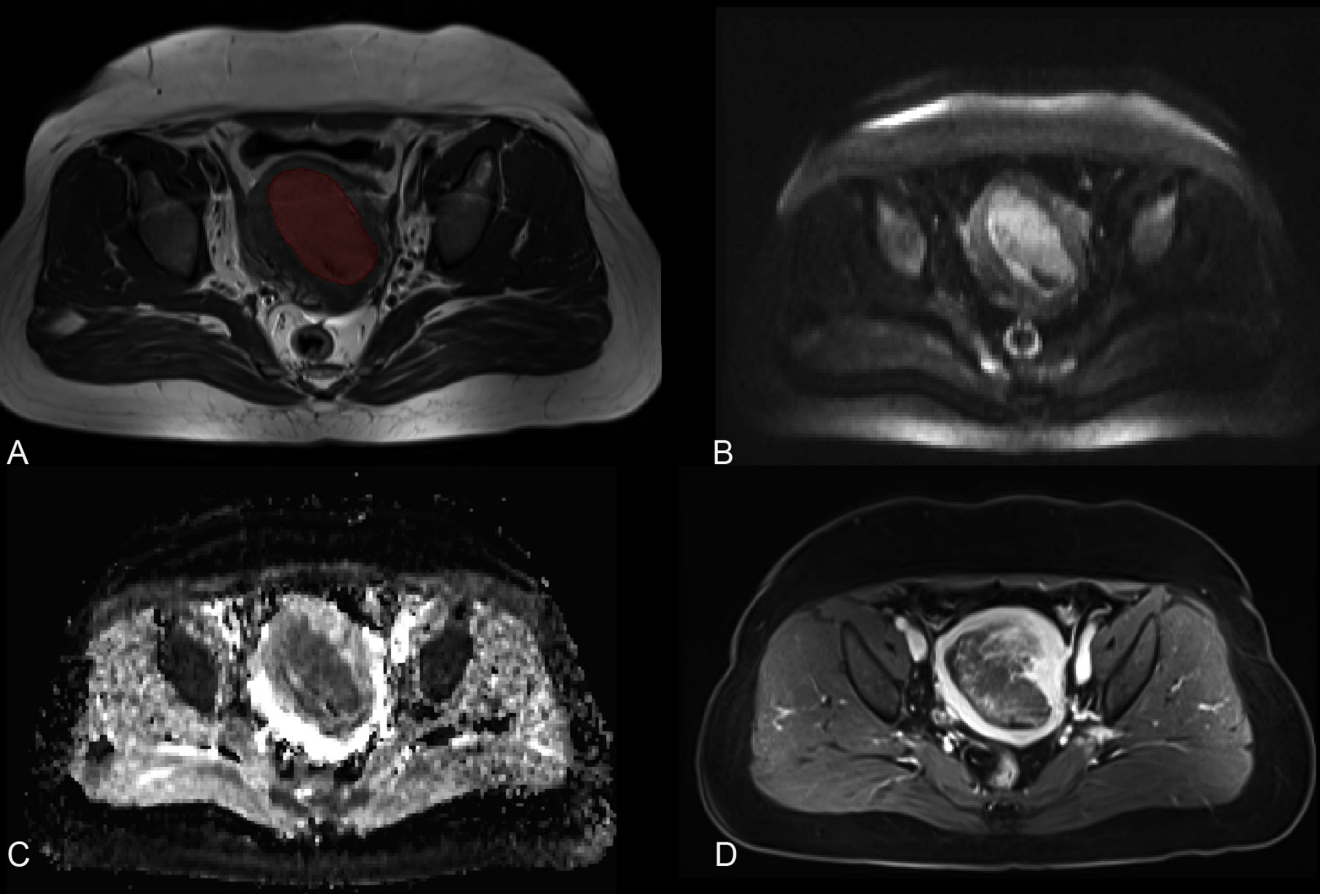
MR images of a 78-year-old woman with endometrial cancer. **(A)** Axial T2-weighted imaging is marked with a region of interest. **(B)** Axial diffusion-weighted imaging (b = 800 s/mm^2^). **(C)** Axial apparent diffusion coefficient imaging. **(D)** Axial contrast-enhanced T1-weighted imaging.

All imaging was delivered from the PACS system and stored in DICOM format. An ROI was drawn around the visible tumor boundary manually in MITK Workbench (http://mitk.org/wiki/The_Medical_Imaging_Interaction_Toolkit(MITK)). The ROIs were drawn based on T2WI cross-sectionally and then referred to the T1WI, DWI (the tumor area shown as high signal in high b value sequences), ADC (the tumor area shown as low signal), and CE-T1WI (delayed phase). All ROIs were drawn by one radiologist (with a 6-year experience in pelvic imaging), who was blinded to the pathological diagnosis, and were rechecked by the other two radiologists (with 32 and 14 years of experience in pelvic imaging, respectively). Thirty days later, 50 patients were randomly selected and the tumors were redrawn by radiologist 1 and by radiologist 2 independently. The ROIs were drawn on each cross-sectional area to generate the volumetric region of interest (VOI, resampled to as isotropic voxels [3 × 3 × 3 mm]) (**Figure 2**).

### Radiomics feature extraction and selection

All the radiomics feature-extracting processes were performed in the Pyradiomics software (https://pypi.org/project/pyradiomics/). Radiomics features based on the VOIs of T1WI T2WI, DWI, ADC, and CE-T1WI were extracted. The images were normalized by subtracting from mean values and dividing by the standard deviation. A voxel array shift of 300 was used to make the gray-level values within a 0–600 range.

To eliminate the effect of different MRI scanning protocols and to improve the classification efficiency of the diagnostic models, a compensation method named “Combat” was used to realign feature distributions computed from different MRI equipment and protocols (16).

The inter- and intraclass correlation coefficients (ICCs) of the extracted features were calculated to assess the reproducibility of radiomics features. Features with both inter- and intraclass ICCs less than 0.75 were considered non-stable features and eliminated. Pearson’s correlation was used to identify redundant features. If two features had a Pearson’s correlation coefficient 0.9, the one with a larger mean absolute coefficient was eliminated.

The Synthetic Minority Oversampling Technique (SMOTE) method was used because of unbalance of positive/negative LVSI samples in the training group. Positive LVSI (minority class) was oversampled and negative LVSI (majority class) was undersampled to balance the training set to improve the classification performance.

Then, a Least Absolute Shrinkage and Selection Operator (LASSO) was used to select non-zero coefficient features associated with LVSI with 10-fold cross-validation in early-stage EC patients. A radscore was calculated for each patient by linear combination of the final selected features.

### Radiomics nomogram build and validation

Clinical parameters of LVSI including age, tumor size, and CA125 were analyzed by using multivariate logistic regression. Clinical risk factors were identified if the parameters were statistically significant in the analysis. A radiomics nomogram was constructed by using multivariable logistic regression analysis which combined the selected radiomics features and the independent clinical risk factors. To assess the discrimination performance of the radiomics nomogram, the area under the curve (AUC) of the receiver operating characteristic (ROC) curve was calculated. Calibration curves were plotted to evaluate the calibration performance of the radiomics nomogram.

The performances of the radiomics signature, clinical risk factors, and radiomics nomogram were validated in the test group. The sensitivity, specificity, and AUC were also calculated.

### Clinical usefulness

By quantifying the net benefits at different threshold probabilities in the training group, decision curve analysis (DCA) was performed to determine the clinical usefulness of the radiomics nomogram.

### Statistical analysis

Statistical analysis was performed by using R software (version 4.0.5; http://www.Rproject.org). An independent sample t-test was used to compare the differences in continuous variables (age, tumor size, and CA125). Chi-squared test was used to compare the differences in categorical variables (FIGO stage, LVSI status). The association between LVSI and clinical risk factors was assessed using Spearman’s correlation. The “glmnet” package was used for LASSO and logistic regression, the “ComBatHarmonization” package was used for Combat, the “DMwR” package was used for SMOTE, the “rms” package was used for nomogram calculation, “pROC” package was used for AUC, and the “dca.R” package was used for DCA. ROC curve analysis was performed to calculate the AUC and corresponding 95% confidence interval. DeLong test was used to compare the performance of clinical risk factors, radscore, and nomogram. A P value less than 0.05 was considered statistically significant.

## Results

### Patients

Three hundred thirty-nine patients were finally included with 54 positive LVSI and 285 negative LVSI (mean age, 56.8 years; range, 25–89 years). All patients underwent total hysterectomy and bilateral salpingo-oophorectomy. Among these, 216 were G1, 75 were G2, and 27 were G3; 277 were superficial or no myometrial invasion, and 62 were deep myometrial invasion. The patients’ clinical risk factors as well as the stage and tumor characteristics of each subgroup are presented in **Table 1**.

**Table 1:** The comparisons of clinicopathologic characteristics between negative and positive LVSI patients in the training and test groups.

Clinical features	Training group			Test group		
	Negative LVSI (N = 188)	Positive LVSI (N = 38)	P	Negative LVSI (N = 97)	Positive LVSI (N = 16)	P
Radscore	0.12 (0.16)	0.39 (0.17)	0.001	0.11 (0.13)	0.31 (0.18)	0.001
CA125	19.3 (13.1)	34.4 (29.1)	0.003	19.5 (13.3)	38.4 (37.3)	0.062
Age	56.0 (8.93)	59.9 (9.44)	0.022	56.1 (8.71)	62.6 (11.9)	0.048
Tumor size	15.3 (5.53)	20.6 (8.62)	0.001	15.4 (3.99)	19.0 (5.57)	0.023
FIGO						
IA	165 (87.8%)	23 (60.5%)	0.001	79 (81.4%)	10 (62.5%)	0.166
IB	23 (12.2%)	15 (39.5%)		18 (18.6%)	6 (37.5%)	
Tumor type						
Carcinosarcoma	1 (0.5%)	1 (2.6%)	0.052	0 (0%)	1 (6.3%)	0.081
Clear cell carcinoma	1 (0.5%)	1 (2.6%)		2 (2.1%)	0 (0%)	
Endometrioid adenocarcinoma	179 (95.2%)	31 (81.6%)		93 (95.9%)	15 (93.8%)	
Mixed adenocarcinoma	2 (1.1%)	2 (5.3%)		0 (0%)	0 (0%)	
Serous adenocarcinoma	5 (2.7%)	3 (7.9%)		2 (2.1%)	0 (0%)	
Tumor grade			0.001			0.154
G1	129 (68.6%)	15 (39.5%)		64 (66.0%)	8 (50.0%)	
G2	42 (22.3%)	8 (21.1%)		22 (22.7%)	3 (18.8%)	
G3	8 (4.3%)	8 (21.1%)		7 (7.2%)	4 (25.0%)	
Others	9 (4.8%)	7 (18.4%)		4 (4.1%)	1 (6.3%)	
Myometrial invasion						
Non-MI and SMI	165 (87.8%)	23 (60.5%)	0.001	79 (81.4%)	10 (62.5%)	0.166
DMI	23 (12.2%)	15 (39.5%)		18 (18.6%)	6 (37.5%)	

CA125, cancer antigen 125; DMI, deep myometrial invasion; LVSI, lymph-vascular space invasion, SMI, superficial myometrial invasion.

### Feature selection and radiomics signature construction

**Figure 3:**
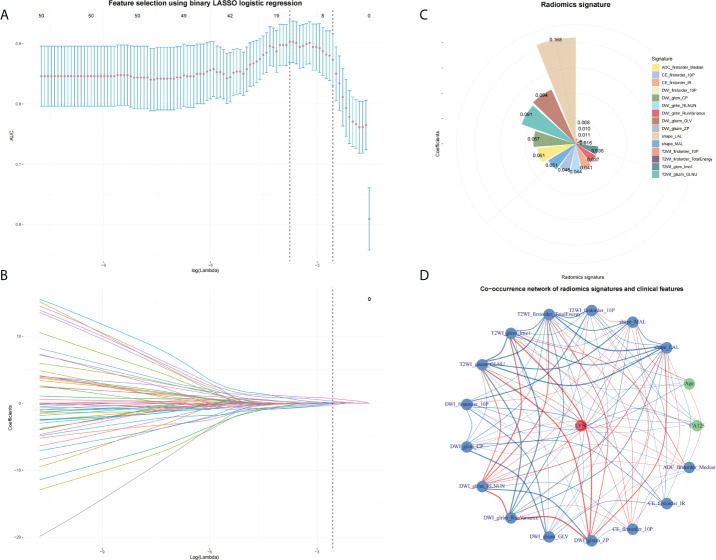
Feature selection by using LASSO. **(A)** The parameter lambda is chosen using 10-fold cross-validation *via* minimum criteria, which results in 10 features with non-zero coefficients. **(B)** LASSO coefficient profiles of the selected features. **(C)** Radiomics signature. **(D)** A co-occurrence network shows the correlations between radiomics signature and clinical features.

A total of 358 radiomics features were extracted, including 14 shape features, 72 first-order features, and 272 texture features. After removing features with either inter- and intraclass ICC 0.75 and Pearson’s correlation coefficients 0.9, 231 and 96 features were retained, respectively. LASSO analysis finally included 15 radiomics features, which were defined as the radiomics signatures (**Figure 3**). The radscore calculation was as follows:

Radscore = 0.16814 + 0.16823×shape_LAL + -0.05128×shape_MAL + 0.00899×T2WI_firstorder_10P + 0.01112×T2WI_firstorder_TotalEnergy + 0.03609×T2WI_glcm_Imc1 + -0.09119×T2WI_glszm_GLNU + -0.01004×DWI_firstorder_10P + -0.0668×DWI_glcm_CP + -0.04433×DWI_glrlm_RLNUN + -0.03675×DWI_glrlm_RunVariance + 0.09361×DWI_glszm_GLV + 0.01592×DWI_glszm_ZP + 0.04461×CE_firstorder_10P + -0.04113×CE_firstorder_IR + -0.06052×ADC_firstorder_Median

### Radiomics nomogram development and validation

**Figure 4:**
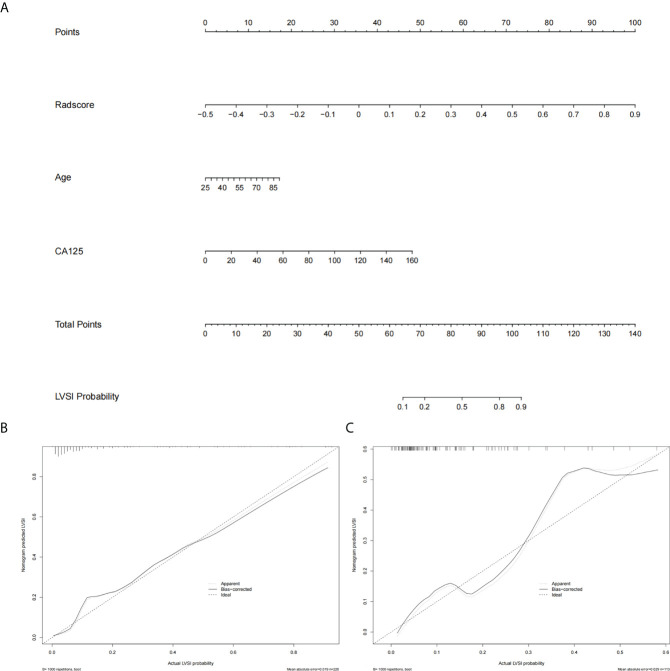
The radiomics nomogram and calibration curves. **(A)** The radiomics nomogram is constructed by integrating radscore with patient age and CA125. The calibration curve of the radiomics nomogram for predicting LVSI in the training group **(B)** and the test group **(C)**.

Multivariate logistic regression analysis showed that age, CA125, and tumor size were risk factors of LVSI in the early-stage EC. On considering that the selected feature “shape_LAL” and “shape_MAL” were the same as tumor size, we did not include tumor size in the nomogram for avoiding overfitting. Therefore, a radiomics nomogram was constructed by integrating the radiomics signatures, age, and CA125 (**Figure 4**).

### LVSI prediction performance

The prediction performance of radscore was 0.88 (95% CI: 0.81–0.94) and 0.82 (95% CI: 0.72–0.93) in the training group and test group, respectively. The prediction performance of clinical risk factors was 0.72 (95% CI: 0.63–0.81) and 0.71 (95% CI: 0.57–0.86) in the training group and test group, respectively. The prediction performance of the radiomics nomogram was 0.89 (95% CI: 0.83–0.95) and 0.85 (95% CI: 0.75–0.94) in the training group and test group, respectively. The AUC, specificity, sensitivity, positive predictive value, and negative predictive values are shown in **Table 2**.

**Table 2:** Diagnostic performance of the clinical risk factors, radscore, and radiomics nomogram in the training and test groups.

Group	Index	AUC	95% CI	SPE	SEN	NPV	PPV	P *	P #
Training	Clinical risk factors	0.72	0.63-0.81	0.71	0.68	0.92	0.32	0.002	–
	Radscore	0.88	0.81-0.94	0.74	0.92	0.98	0.42	–	0.002
	Nomogram	0.89	0.83-0.95	0.76	0.92	0.98	0.43	0.254	0.001
Test	Clinical risk factors	0.71	0.57-0.86	0.81	0.56	0.92	0.33	0.239	–
	Radscore	0.82	0.72-0.93	0.94	0.56	0.93	0.60	–	0.239
	Nomogram	0.85	0.75-0.94	0.96	0.56	0.93	0.69	0.116	0.104

AUC, area under the curve; CI, confidence interval; NPV, negative predictive value; PPV, positive predictive value; SEN, sensitivity; SPE, specificity.

*Compared with radscore; # compared with clinical risk factors by DeLong test.

### Clinical usefulness

**Figure 5:**
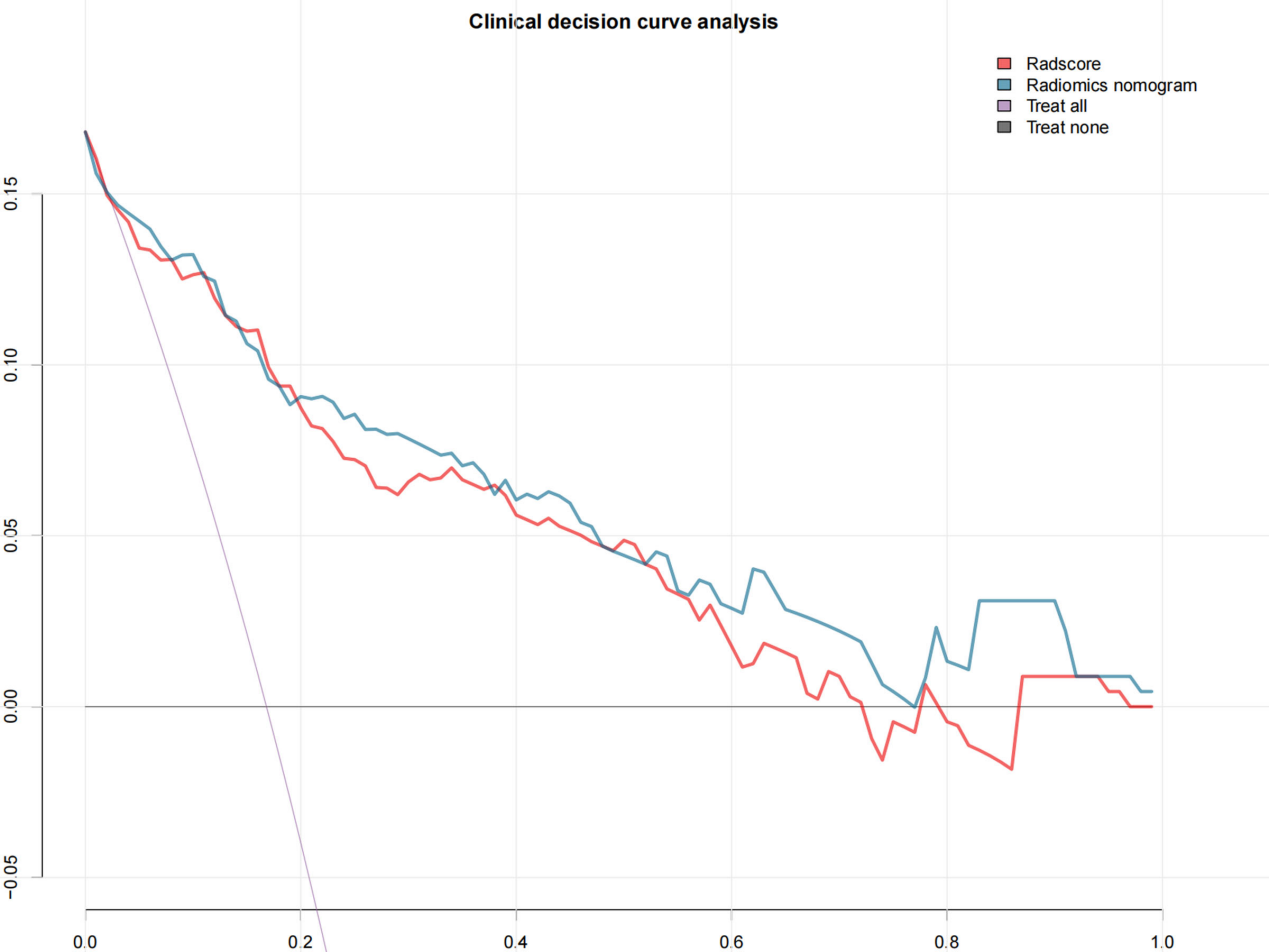
The decision curve shows that when the threshold probability is from 20% to 100%, the radiomics nomogram adds more net benefit than schemes of treat-all, treat-none, and radscore.

The DCA showed that both the radiomics signatures and radiomics nomogram could add net benefit to the patients to predict the presence of LVSI preoperatively (**Figure 5**). When the threshold probability was within a range from 20% to 100%, the net benefit of using the nomogram to predict LVSI was more than the treat-all or treat-none scheme.

## Discussion

Our preliminary study showed that radiomics features based on the multiparamater MRI image had high diagnosis performance for LVSI in early-stage EC. It indicated that this computer-based data analysis could be used as a helpful diagnosis tool to predict the presence of LVSI.

Previous studies showed that the ADC value could differentiate the presence of LVSI with moderate SEN and SPE of 65% and 80%, respectively (17). This was because limited information could be provided by the quantitative analysis based on functional MRI. Moreover, analyzing the characteristics of EC based on a single MRI sequence also had limitations. The heterogeneity of EC was not only reflected in the restricted water molecular movement in the DWI or ADC sequence. More information could be also provided by other MRI sequences such as CE sequences (18). According to our results, a good diagnosis performance was found in radiomics features extracted from the T2WI, DWI, ADC maps, and CE-T1WI sequences for predicting the presence of LVSI. This might be because the radiomics features could provide information in unraveling the tumor heterogeneity, which could not be captured by human eyes (13, 19).

The diagnosis performance of the radiomics nomogram was higher than the previously reported study. Yoshiko et al. suggested that the texture feature based on tumor largest slice analysis could differentiate the absence of LVSI with AUC, SEN, and SPE of 0.80, 80.9%, and 72.5%, respectively (11). The reason may be because we extracted and included the features from high-dimensional data. Yoshiko et al.’s study only included the first-order features (11). Results indicated that higher-order features were more helpful to predict the presence of LVSI. Furthermore, our analysis was based on the tumor volume rather than on the largest tumor layer. The tumor volume may contain more information representative of the tumor heterogeneity, which may produce more accuracy information of the tumor.

Significantly higher tumor grade, deep myometrial invasion, and larger tumor size were shown in LVSI patients. This suggested that patients with higher tumor grade, deep myometrial invasion, and larger tumor size were more likely to have a positive LVSI. These results were in accordance with previous studies (20). Larger tumor size was significantly and independently associated with LVSI and myometrial invasion in patients with early-stage EC (16, 20). Given the difficulty of obtaining reliable LVSI data from frozen sections, tumor size might be used as a surrogate at the time of surgery to provide additional information to triage patients for treatment (8).

Disputes existed in whether lymphadenectomy should be performed in early-stage EC patients (21). On considering that LVSI was an independent risk factor for developing pelvic lymph node metastasis in early-stage EC, a preoperative evaluation of LVSI was of clinical use in the decision-making process before performing a lymphadenectomy in early-stage EC patients (22). The LVSI could not be assessed by the human eyes; radiomics could provide useful preoperative information from standard MR images instead, and it may help physicians to grade the patients’ risk levels and to guide appropriate treatment.

The strengths of this study were as follows: This was a multicenter study with large samples. The ComBat method was used to remove variation before combining data across MRI scanners and sites. Also, our study had same limitations. First, shortcomings of the inherent retrospective study should be considered. Second, high-order wavelet or Guess features were not included for the reason that these features may not be stable and lack reasonable clinical interpretation. Third, the imbalance between negative and positive LVSI should be noticed. However, a sample equilibrium algorithm was used in the training progress; no obvious diagnostic performance was declined in the test group. Fourth, the differences in segmentations were not checked, and it is possible that some radiomics features that would have been robust might be discarded because of extracting from largely different volumes. However, the radiomics nomogram model performed well in both the training and test cohorts.

In conclusion, the radiomics nomogram could individually predict LVSI in patients with early-stage EC. The nomogram could be conveniently used to facilitate the treatment decision for clinicians.

## Data availability statement

The raw data supporting the conclusions of this article will be made available by the authors, without undue reservation.

## Ethics statement

The studies involving human participants were reviewed and approved by Institutional review board of Obstetrics & Gynecology Hospital, Fudan University. Written informed consent for participation was not required for this study in accordance with the national legislation and the institutional requirements.

## Author contributions

B-CY and F-HM designed the research study. X-FL, B-CY and YL performed the research. B-CY and F-HM provided help and advice on acquisition of data. YL and X-FL analyzed the data. YL and X-FL wrote the manuscript. YL and J-WQ were the supervisors of this study. All authors read and approved the final manuscript.

## Funding

This research was funded by Shanghai Science and Technology Committee (No. 20JC1418200).

## Conflict of interest

The authors declare that the research was conducted in the absence of any commercial or financial relationships that could be construed as a potential conflict of interest.

## Publisher’s note

All claims expressed in this article are solely those of the authors and do not necessarily represent those of their affiliated organizations, or those of the publisher, the editors and the reviewers. Any product that may be evaluated in this article, or claim that may be made by its manufacturer, is not guaranteed or endorsed by the publisher.
