# Supplementary material for: Radiomics feature as a preoperative predictive of lymphovascular invasion in early-stage endometrial cancer: A multicenter study
Source: Front Oncol. 2022 Aug 18;12:966529. doi: 10.3389/fonc.2022.966529 (PMC9433783; doi:10.3389/fonc.2022.966529)
Supplement: Supplementary file 1 [file Table_1.docx]

Supplementary Table 1. MRI examination's parameters

| Characters | Center A | Center B | Center C | Center D | Center E |
| --- | --- | --- | --- | --- | --- |
| MRI | 1.5-T Avanto Siemens Erlangen Germany | 3.0-T Trio Siemens Erlangen Germany | 3.0-T Verio Siemens Erlangen Germany | 1.5-T OPTIMA 360 General Electric America | 3.0-T Verio Siemens Erlangen Germany |
| T1WI | TSE,TR/TE=761/10,Matrix=512×512, thickness = 4mm, FOV = 360×280mm | TSE,TR/TE=700/11,Matrix=512×512, thickness = 5mm, FOV = 380×280mm | TSE,TR/TE=340/10,Matrix=512×512, thickness = 4mm, FOV = 340×280mm | FSE,TR/TE=788/15,Matrix=512×512, thickness = 4mm, FOV = 350×270mm | VIBE,TR/TE=3.4/1.3,Matrix=320×320, thickness = 3mm, FOV = 380×280mm |
| T2WI | SE,TR/TE=4000/98 or 8000/83,Matrix=512×512, thickness = 4mm, FOV = 360×280mm | SE,TR/TE=3300/88,Matrix=512×512, thickness = 3mm, FOV = 380×280mm | TSE,TR/TE=8000/83,Matrix=512×512, thickness = 4mm, FOV = 340×270mm | FSE,TR/TE=2705/60,Matrix=512×512, thickness = 4mm, FOV = 380×270mm | TSE,TR/TE=2770/64,Matrix=320×320 , thickness = 4mm, FOV = 380×280mm |
| DWI | EPI,TR/TE=4000/100,b=800(or1000),Matrix=256×256, thickness = 5mm, FOV = 360×80mm | EPI,TR/TE=2500/100,b=800(or1000),Matrix=256×256, thickness = 4mm, FOV = 380×280mm | EPI,TR/TE=4000/100,b=1000,Matrix=256×256, thickness = 5mm, FOV = 340×280mm | EPI,TR/TE=3708/76,b=800,Matrix=256×256, thickness = 5mm, FOV = 380×270mm | EPI,TR/TE=7100/79,b=1000,Matrix=256×256, thickness = 4mm, FOV = 380×280mm |
| CE-T1WI | FLASH,TR/TE=196/2.9,Matrix=512×512, thickness = 4mm, FOV = 360×280mm | TSE,TR/TE=700/11,Matrix=512×512, thickness = 5mm, FOV = 380×280mm | TSE,TR/TE=196/2.9,Matrix=512×512, thickness = 4mm, FOV = 340×280mm | LAVA,TR/TE=3.7/1.7,Matrix=512×512, thickness = 4mm, FOV = 350×270mm | VIBE,TR/TE=3.4/1.3,Matrix=320×320, thickness = 3mm, FOV = 380×280mm |

CE: contrast enhanced; DWI: diffusion weighted imaging; EPI: echo planar imaging; FLASH: fast low angle shot sequence; FOV: field of view; FSE: fast spin echo; LAVA: liver acquisition with volume acceleration; MRI: magnetic resonance imaging; SE: spin echo; T1WI: T1 weighted imaging; T2WI: T2 weighted imaging; TE: echo time; TR: repetition time; TSE: turbo spin echo; VIBE: volumetric interpolated breath-hold examination
